# Precision medicine: a new era for inner ear diseases

**DOI:** 10.3389/fphar.2024.1328460

**Published:** 2024-01-24

**Authors:** Elisa Tavazzani, Paolo Spaiardi, Donatella Contini, Giulio Sancini, Giancarlo Russo, Sergio Masetto

**Affiliations:** ^1^ Department of Molecular Medicine, University of Pavia, Pavia, Italy; ^2^ ICS-Maugeri IRCCS, Pavia, Italy; ^3^ Department of Biology and Biotechnology “Lazzaro Spallanzani”, University of Pavia, Pavia, Italy; ^4^ Istituto Nazionale di Fisica Nucleare, Sezione di Pavia, Pavia, Italy; ^5^ Department of Anatomy and Cell Biology, University of Illinois at Chicago, Chicago, IL, United States; ^6^ Department of Medicine and Surgery, University of Milano-Bicocca, Milan, Italy; ^7^ Nanomedicine Center, Neuroscience Center, School of Medicine and Surgery, University of Milano-Bicocca, Milan, Italy; ^8^ Department of Brain and Behavioral Sciences, University of Pavia, Pavia, Italy

**Keywords:** vestibule, cochlea, inner ear, nanoparticles, microneedles, precision medicine

## Abstract

The inner ear is the organ responsible for hearing and balance. Inner ear dysfunction can be the result of infection, trauma, ototoxic drugs, genetic mutation or predisposition. Often, like for Ménière disease, the cause is unknown. Due to the complex access to the inner ear as a fluid-filled cavity within the temporal bone of the skull, effective diagnosis of inner ear pathologies and targeted drug delivery pose significant challenges. Samples of inner ear fluids can only be collected during surgery because the available procedures damage the tiny and fragile structures of the inner ear. Concerning drug administration, the final dose, kinetics, and targets cannot be controlled. Overcoming these limitations is crucial for successful inner ear precision medicine. Recently, notable advancements in microneedle technologies offer the potential for safe sampling of inner ear fluids and local treatment. Ultrasharp microneedles can reach the inner ear fluids with minimal damage to the organ, collect μl amounts of perilymph, and deliver therapeutic agents *in loco*. This review highlights the potential of ultrasharp microneedles, combined with nano vectors and gene therapy, to effectively treat inner ear diseases of different etiology on an individual basis. Though further research is necessary to translate these innovative approaches into clinical practice, these technologies may represent a true breakthrough in the clinical approach to inner ear diseases, ushering in a new era of personalized medicine.

## 1 Background

**FIGURE 1:**
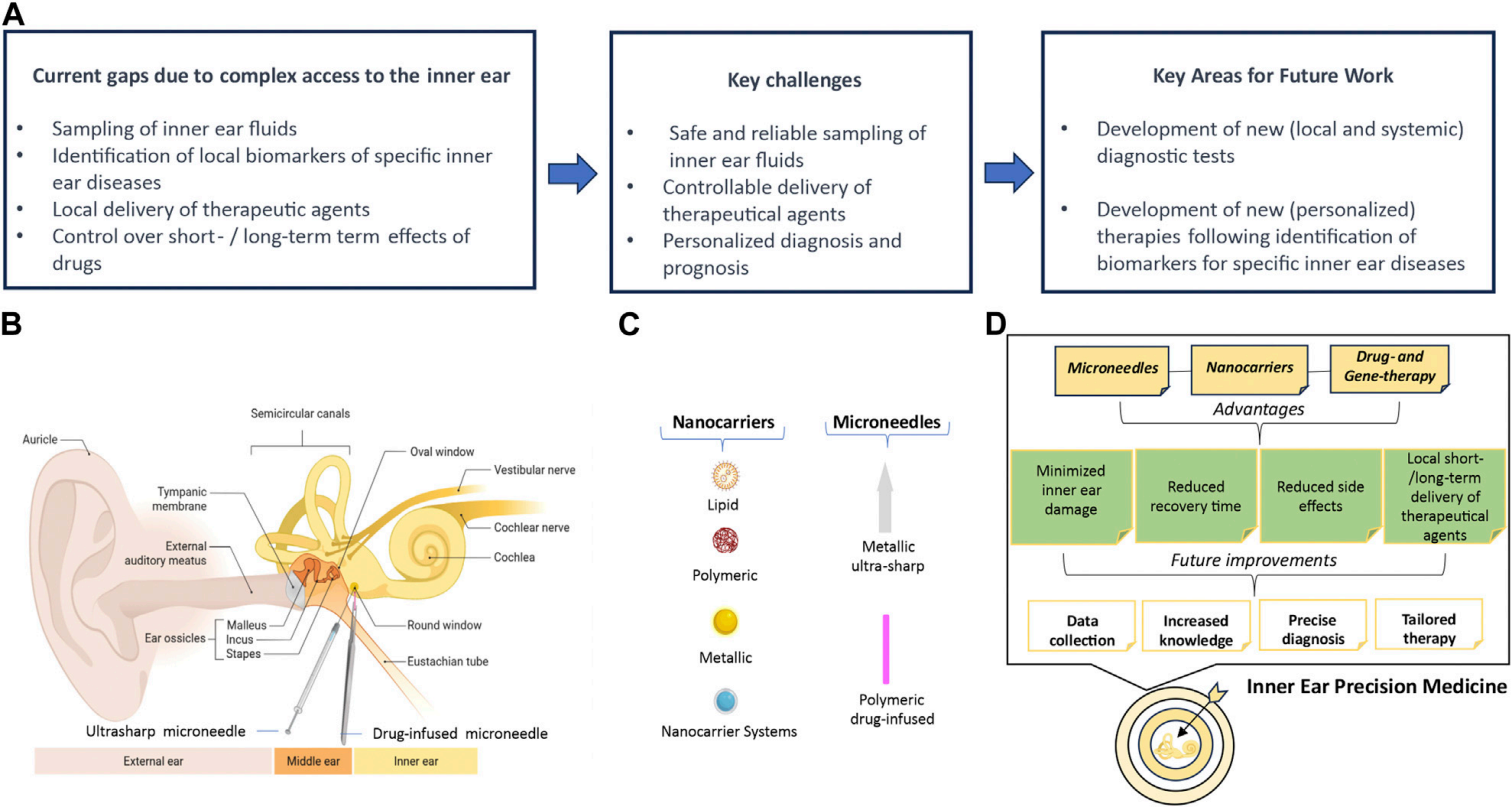
Essential features for precision medicine in inner ear. **(A)** Current gaps, goals to reach in the near future, and future aims. **(B)** Schematic drawing of the human ear showing the RWM approach for two types of microneedles (ultrasharp and PLGA). The RWM separates the middle ear from the inner ear. The inner ear contains the cochlea, the auditory organ, and the vestibular system, the balance organ. This is composed of the utricle and saccule, which detect head position and linear acceleration, and the three semicircular canals, which detect head angular accelerations. **(C)** The most common types of nanocarriers and microneedles that can be used for drug delivery in the inner ear. **(D)** Road map to precision medicine in the inner ear. The panels **B** and **C** were created with BioRender 2024. Licence numbers: TP26BU1SAY, SR26BU1Z32.

The ear is anatomically subdivided into three parts: outer, middle, and inner ear (Figure 1). The outer part includes the auricle and the external auditory canal, and its function is to convey the acoustic vibrations to the 105 µm-thick tympanic membrane (Hentzer, 1969; Hayes et al., 2013; Dsouza et al., 2018). The middle ear transmits the vibration of the tympanic membrane to the oval window membrane of the inner ear via the malleus, incus and stapes. Like the outer ear, the middle ear contains air since it is connected to the nasopharynx by the Eustachian tube. In contrast, the inner ear is filled with fluids and houses the cochlea, the auditory organ, and the vestibular system, the equilibrium organ. Within the cochlea, the *scala tympani* and *scala vestibuli* are connected to each other through the helicotrema at the apex of the cochlea and are filled with perilymph, a standard Na^+^-based extracellular solution; the *scala media*, or the middle chamber contains endolymph, a unique extracellular solution characterized by high K^+^ and low Ca^2+^ and Na^+^ concentration (Wangemann et al., 2017). The *scala media* is connected to the vestibular system via the *ductus reuniens*. Additionally, near the round window membrane (RWM), the *scala tympani* is connected to the cochlear aqueduct, which is connected to the subarachnoid space filled with cerebrospinal fluid. The sensory function in the inner ear is carried out by sophisticated sensory cells: the hair cells (HCs), which are equipped with a tuft of long microvilli at their apical surface, called stereocilia. The apical region of the HCs is bathed by the endolymph, while their basolateral region is bathed by the perilymph. Endolymph motion induced by acoustic vibrations or head movements deflects the stereocilia of cochlear or vestibular HCs, thereby opening mechanosensitive cation channels. The resulting depolarization opens voltage-gated Ca^2+^ and K^+^ channels expressed in the HC basolateral membrane. Ca^2+^ inflow triggers glutamate exocytosis onto the afferent nerve fibers, while K^+^ outflow repolarizes the HC. Signals are then transmitted by the primary neurons to the central nervous system for processing.

Inner ear disorders are the most common sensory pathologies worldwide and include deafness, sensorineural hearing loss, Ménière’s disease, benign paroxysmal positional vertigo, labyrinthitis, secondary endolymphatic hydrops, and perilymphatic fistula. The World Health Organization estimates that 5.5% of the world’s population experiences moderate or high hearing loss and that by 2050, 1 in 10 people will have a disabling hearing deficit (World Health Organization, 2021). Additionally, the incidence of balance disorders of peripheral vestibular origin increases after the age of 40 and exceeds 80% of people over 80 years of age (Agrawal et al., 2009). Ménière’s disease alone affects 2 over 1,000 people (Alexander and Harris, 2010; Koenen and Andaloro, 2021). This pathology causes devastating vertigo attacks (Yardley et al., 2003) and standard treatment with gentamicin leaves patients with permanent cochlear deficits (Blakley, 2000; Harner et al., 2001). Impairment of inner ear function severely affects work and social life (Quaranta et al., 2015). The importance of auditory and vestibular functions in daily activities, the widespread incidence of hearing loss and vestibular dysfunction, and the lack of selective inner ear sampling for accurate diagnosis and therapies provide a strong clinical and ethical motivation to expand the research in this field.

## 2 Major gaps in diagnosis and therapy of inner ear diseases

The location of the inner ear in the osseous labyrinth, the densest and hardest human bone (Frisch et al., 1998), greatly complicates perilymph sampling, which is required for a timely and precise diagnosis, and local drug delivery.

When a patient has an acute or chronic illness of the inner ear, it is likely to be reflected by abnormalities in the presence or concentration of various ions, proteins, bacteria, or viruses when compared to healthy perilymph. Clinically, this limitation results in a frequent diagnosis of idiopathic hearing/vestibular disease, leaving the patient and physician with no clear prognosis and general, and often ineffective, treatment options.

Current procedures for inner ear fluid sampling use glass capillaries, which do not perforate the RWM and therefore can only be used in patients already under surgery, e.g., for removal of acoustic neuromas or cochlear implantation (Lysaght et al., 2011).

Concerning treatment, the main routes of drug administration to the inner ear are oral, intravenous, intracochlear and intratympanic.

Oral is the simplest way, while intravenous requires medical staff. Both allow for only small lipophilic drugs to be administered because the blood-labyrinth barrier precludes the passage of high-molecular-weight drugs. As a result, only a very low fraction reaches the inner ear (Parnes et al., 1999; Bird et al., 2007). Therefore, either a relatively large amount of drug is needed, which increases the importance of side effects, or the treatment is only minimally effective (Nakashima et al., 2003; Ishiyama et al., 2017).

Intracochlear administration is used in preclinical studies to test the safety of drug candidates and has been adapted to different formulations: liquid, suspension, gel (De Ceulaer et al., 2003; Paasche et al., 2006; Braun et al., 2011). It has the advantage of achieving long-term local drug delivery (weeks or even years). The drawback is the low injectable volume, few µL (Braun et al., 2011), because even slight volume and pressure changes can harm the fragile inner ear organs (Jaudoin et al., 2021). Moreover, it requires surgery under general anesthesia performed by highly specialized personnel (El Kechai et al., 2015a). For these reasons, the intracochlear route is not generally used in humans.

The fourth route, intratympanic administration, is widely used for pharmacological treatment in Ménière’s disease (Patel, 2017) due to its minimal invasiveness and compatibility with repeated injections of liquid, suspension, or hydrogel formulations. Intratympanic administration uses a long and fine needle, spinal puncture needles from 22 to 25 G in human (Herraiz et al., 2010) and from 27 to 30 G in animals (Wang et al., 2009; Salt et al., 2011). Then, a volume of 0.4–0.6 mL in humans (Herraiz et al., 2010) or 50–200 μL in animals (Wang et al., 2009) of drug solution is injected, filling the middle ear cavity and ensuring that the drug is in contact with the RWM (El Kechai et al., 2015b). One problem with this approach is that the hole produced in the tympanic membrane is a potential entry point for pathogens. The drug is absorbed into the inner ear perilymph primarily through the semi-permeable RWM, but also via the oval window annular (Phillips and Westerberg, 2011). Since the injected drug must diffuse through the middle ear to reach the RWM, some loss of the medicine occurs through the Eustachian tube (Ramaswamy et al., 2017), resulting in highly variable medication levels among patients. Direct placement of therapeutic agents on the RWM in a biodegradable carrier substance, such as gelatin, hydrogel, or nanoparticles, may overcome some of these limitations (Paulson et al., 2008; Li et al., 2012; Zhang et al., 2018). However, the rate of drug delivery to the inner ear is inevitably limited by molecular diffusion across the RWM. In summary, the reliability of intratympanic administration is severely impacted by the variable diffusion of the drug through the middle ear and the RWM.

Given the above, it is evident the need for safe and reliable access to the inner ear for both diagnosis and treatment purposes.

## 3 Microneedles may help filling the gaps

Without a means to sample inner ear fluid for electrochemical, genetic, or proteomic analysis, precise intervention is not possible. Furthermore, current options for drug delivery, including systemic administration and intratympanic injection, are imprecise. The only access from the middle ear to the inner ear that does not require perforation of bone is through the RWM. The human RWM has a surface area of 2.3 mm^2^ (Okuno and Sando, 1988) and a thickness of 70 µm (Goycoolea and Lundman, 1997). The distance between the human RWM and the basilar membrane—the nearest structure within the inner ear—is approximately 1.2 mm (Paprocki et al., 2004; Watanabe et al., 2016). The primary risks associated with RWM perforation concern damage to the fragile structures of the inner ear, homeostasis impairment due to perilymph leakage and external contamination. Fortunately, recent technical advances in microneedle architecture and manufacturing have minimized the negative consequences of RWM perforation, also reviewed in Leong et al. (2022). Chiang et al. (2020), manufactured 3D-printed microneedles able to create small (μm scale) holes in cadaveric human RWM without damaging the cochlea. 3D printing relies on a technology called two-photon polymerization lithography, an additive manufacturing process that can produce highly complex geometries out of hard polymers with sub-micrometer precision and accuracy. Fully-metallic (copper) ultra-sharp microneedles were conversely developed by Aksit and colleagues (Aksit et al., 2021), which were gold-coated to ensure biocompatibility. Minimal trauma was observed in both guinea pig and human cadaveric RWMs. Thus, microneedle RWM perforation might be performed prior to intratympanic delivery to overcome variable diffusion of the therapeutic agent through the RWM (Szeto et al., 2019). Concerning perilymph sampling, Early and colleagues (Early et al., 2019) developed a silver-plated hollow microneedle device for trans-RWM liquid biopsy, by which 1 μL of perilymph from post-mortem human temporal bones could be obtained without damaging the inner ear structures. This is a notable result when considering that the whole perilymph per ear is about 150 μL (Leong et al., 2023a). By the same strategy, Leong and colleagues (Leong et al., 2023a) very recently developed a 3D-printed hollow microneedle for diagnostic aspiration of perilymph and intracochlear delivery of therapeutic agents in living guinea pigs. Perforation did not cause hearing loss, healed within 48–72 h, and yielded sufficient perilymph for proteomic analysis. A slightly different approach has been used by Pawley and colleagues (Pawley et al., 2021), who developed a drug-infused polymeric microneedle designed for penetrating the RWM and resting in the base of the scala tympany of the rat cochlea, to deliver a uniform dose of the drug over an extended period (about 50% of drug was released in 1 month). Microneedles were made of poly (lactic-co-glycolic acid); (PLGA), a biodegradable polymeric nanoparticle (see next section) approved by the Food and Drug Administration. Their microneedles had over ∼4,000× the mechanical strength required to puncture rodent RWM and are therefore suitable for human use. This is the only study reporting delivery of therapeutic agents by microneedles so far.

In summary, microneedle-mediated perforation of the RWM is a novel means of achieving access to the inner ear with minimal anatomic and functional damage. Microneedles are designed to aspirate fluids for diagnosis and to deliver therapeutic agents. Using microneedles, drug concentrations within the inner ear may be controlled with a precision that intratympanic injections cannot provide. Microneedle’s properties are summarized in Table 1. *In vivo*, microneedles have only been tested in rodents so far, although perforation of human RWM has been accomplished *postmortem*. It should be noted that RWM perforation requires surgery of the temporal bone and therefore cannot be considered as a simple routine procedure.

**TABLE 1:** Main properties of microneedles.

Microneedles’ properties
1. The materials used for the fabrication (metals, polymers) are hard and resistant enough to perforate the RWM in both animal models and humans (Early et al., 2019; Szeto et al., 2019; Chiang et al., 2020; Yu et al., 2020; Aksit et al., 2021; Pawley et al., 2021)
2. The manufacturing techniques are highly versatile, allowing for different shapes and diameters of the microneedle tips (Watanabe et al., 2016; Early et al., 2019; Szeto et al., 2019; Yu et al., 2020; Aksit et al., 2021; Leong et al., 2022)
3. The same microneedle can be luminized and used to perforate the RWM and sample or/and deliver therapeutic agents, streamlining the procedure. This eliminates the need for the double procedure involving RWM perforation first, and sampling/delivery by a second device (Szeto et al., 2019; Chiang et al., 2020; Yu et al., 2020; Aksit et al., 2021; Pawley et al., 2021; Leong et al., 2022)
4. The materials employed for the microneedles are biocompatible (Aksit et al., 2021; Pawley et al., 2021; Leong et al., 2023b)
5. The architecture enables control of perforation by detecting the reaching of the perilymph (Wazen et al., 2017; Early et al., 2019)
6. The tip of the microneedles can be designed to smoothly cross the RWM, minimizing damage (reducing healing time) and perilymph leakage in the middle ear (Early et al., 2019; Aksit et al., 2021; Leong et al., 2023a; Feng et al., 2023)
7. The new microneedles can be used for sampling or acute delivering (ultra-sharp microneedle) or implanted for long-term delivery (PLGA microneedles) due to their biodegradable nature (Aksit et al., 2021; Pawley et al., 2021; Leong et al., 2023b; Feng et al., 2023)
8. The tiny dimensions of the microneedles tips allow for sampling/delivering small (μl scale) volumes (Aksit et al., 2021; Leong et al., 2023a; Feng et al., 2023)

## 4 Nanocarriers for drug delivery to the inner ear

The treatment of inner ear disorders may soon benefit from the rapid development of nanocarriers to deliver drugs and genetic material. The obstacles to overcome, besides access to the inner ear as discussed above, are: a) the control for long-term release of the drug; b) the development of side effects; c) the degradation of the carrier/carried agent. The biocompatibility, biodegradability, size, volume, composition, shape and chargeable surface of nanocarriers, their bio-distribution in the target and off-target organs, metabolism and elimination are all important features to be addressed.

The main nanoparticles (NPs) so far considered for inner ear therapy are listed below, categorized for their molecular nature (Figure 1C):

A) Lipid-Based Nanocarriers: Liposomes, Solid Lipid Nanoparticles (SLNs), Nanostructured Lipid Carriers (NLCs).

Liposomes, the first phospholipid vesicle system developed in the 1960s, are composed of phospholipid bilayer similar to the plasma membrane of human cells. They have good biocompatibility and promote drug diffusion across the plasma membrane. Liposomes, with sizes ranging from 20 nm to over 1 μm, typically consist of a hydrophobic bilayer that encapsulates lipophilic drugs and a hydrophilic core for holding and stabilizing hydrophilic drugs. Such features make liposomes a versatile vehicle to carry both hydrophilic and hydrophobic drugs in the aqueous lumen and lipid bilayer respectively. In addition, the liposome system offers the advantage of easy modification and targeting potential. Liposomes can be constructed with their surfaces modified using appropriate molecules or ligands to actively bind a target molecule within specific cells, systems, or tissues (Lu et al., 2021). Recently, liposomes have been used to treat autosomal dominant hearing loss by *in vivo* delivery, through a cannula following cochleostomy, of genome editing agents in transgenic mice (Gao et al., 2018).

SLNs combine the advantages of polymer nanocarriers, including a high drug loading capacity, controllable drug delivery, and good biocompatibility with lipid emulsions, thereby improving drug bioavailability. SLNs can be prepared by using a variety of technologies including heat or cold homogenization, making them easily scalable for production. Due to their small sizes and large surface area, SLNs are suitable to be covered with functionalized ligands moieties, antibodies, and other functional groups (Lu et al., 2021).

NLCs, also known as lipid-based formulations, have been broadly studied as drug delivery systems due to their enhanced physical stability, improved drug loading capacity, and biocompatibility (Haider et al., 2020). Unlike SLNs, the lipid matrix of NLCs consists of a mixture of solid and liquid lipids with controlled levels that have an improved capacity for bioactive retention along with controlled release attributes (Akhavan et al., 2018).

B) Polymeric Nanoparticles: Polymersomes may pass the RWM without toxic effects (Buckiova et al., 2012; Roy et al., 2012); Chitosan is a natural biodegradable and biocompatible polymer with antifungal and antibacterial properties deliverable in the inner ear (No et al., 2002; Saber et al., 2010; Lajud et al., 2015; Khanna et al., 2023; Rezaei Abbas Abad et al., 2023); PLGA NPs are suitable for the treatments of inner ear diseases for their tunable degradation, mechanical properties, drug-infused microneedle fabrication (Lehner et al., 2019; Pawley et al., 2021), and gene therapy (Du et al., 2018).
C) Metallic NPs: Superparamagnetic Iron Oxide Nanoparticles permit a precise drug delivery in the inner ear by magnetic forces (Kopke et al., 2006); Silver Nanoparticles have antifungal, antibacterial, antiviral properties and are suitable in otitis treatment (Zou et al., 2014); Gold Nanoparticles are chemically stable and biocompatible (Blebea et al., 2022); Silica Nanoparticles may serve as a nonviral delivery system to the sensory HCs (Praetorius et al., 2007).
D) Nanocarrier Systems: Nanoparticle-Hydrogel Systems are thermosensitive solidifying in the middle ear for a sustained dose release (Lambert et al., 2016); Cell Penetrating Peptides can be used to deliver cargo into the developing inner ear (Miwa et al., 2011).

For comprehensive reviews classifying nanocarrier biodistribution, pathway mechanisms and drug pharmacokinetics see: Jaudoin et al., 2021; Dindelegan et al., 2022.

## 5 Inner ear and gene therapy

Thanks to recent advances in understanding the genetic basis of several inner ear diseases, delivery of genetic material (DNA, RNA, siRNA, microRNA, antisense oligonucleotides, or CRISPR/Cas9) has emerged as a promising strategy for their treatment (Lentz et al., 2020; Bankoti et al., 2021; Cui et al., 2022; Nacher-Soler et al., 2022). This approach aims to control gene replacement, silencing, augmentation, and editing and it could be an option to treat hearing loss and vestibular disorders (Fukui and Raphael, 2013; Geng et al., 2018; Lentz et al., 2020; Bankoti et al., 2021; Cui et al., 2022; Nacher-Soler et al., 2022). The most common and successful way of delivering genetic material to the inner ear of rodents is through the RWM (Akil et al., 2012; Askew et al., 2015; Pan et al., 2017a; Emptoz et al., 2017; Landegger et al., 2017; Dulon et al., 2018) further highlighting the importance of perfecting this route of administration for future use in humans. Notably, György and colleagues (Gyorgy et al., 2019) recently showed that the RWM approach leads to efficient transgene transfer into the cochlea of non-human primates (Gyorgy et al., 2019). Alternative delivery routes to the inner ear, so far positively tested in rodents, involve injection of agents into the posterior semicircular canal (Okada et al., 2012; Suzuki et al., 2017; Isgrig and Chien, 2018), or into the cerebrospinal fluid of the *cisterna magna*, which is connected to the inner ear by the cochlear aqueduct (Mathiesen et al., 2023). The combination of trans-RWM injection and canalostomy in adult mice has recently been shown to increase the efficiency of gene transduction in cochlear inner HCs in all turns of the cochlea without impairing auditory function or hearing (Yoshimura et al., 2018).

However, several questions need to be answered before gene therapy translation to humans. Due to challenges in maintaining inner ear tissues *in vitro*, tests in human tissues are still limited (Kesser et al., 2007). Unresolved points include differences in cell trophism and chronological maturation between humans and animal models, and strategies to confine gene therapy to target organs. Addressing issues such as negative consequences of gene overexpression/silencing in target and off-target cells, as well as evaluating the long-term safety of exogenous constructs, immune response in case of multiple administrations, time between doses, and risk of infection before and after gene therapy is imperative (Wu et al., 2019).

Delivery routes and vectors depend on material size, cargo capacity, pathogenicity, immunogenicity, and transduction efficiency. There are two main delivery systems for gene therapy: (i) viral vectors: viruses modified and attenuated to create effective and specific tools for gene transfer, and (ii) non-viral delivery/vectors: nanoparticles and microspheres consisting of biodegradable polymers. Adeno-associated viral vectors (AAVs) and synthetic viral vectors (Anc80L65 and AAV2.7m8) are commonly chosen for their effectiveness in the inner ear (Pan et al., 2017b; Isgrig et al., 2017). Non-viral vectors (nanoligand drug carriers self-assembled from a phage display peptide) (Delmaghani and El-Amraoui, 2020) avoiding virus integration into human DNA, represent alternatives due to their easy use, reduced toxicity and immunogenicity compared to viral vectors. Careful consideration of these factors is essential for successful gene therapy applications. For extensive reviews about gene therapy in the inner ear see Delmaghani and El-Amraoui and Chaves & Holt (Delmaghani and El-Amraoui, 2020; Chaves and Holt, 2023). Gene therapy could also take advantage of microneedles for delivery.

## 6 Discussion and future prospects

One of the critical determinants of the success of precision medicine in the inner ear is to find safe and reliable access to perilymph for personalized diagnosis and delivery of pharmacological agents. The new ultrasharp microneedles meet these requirements, and together with nano vectors and gene therapy offer great promise as a potential treatment for human inner ear disease of environmental or genetic cause. In perspective, sampling of inner ear fluids could identify biomarkers of specific diseases that might be also detected in routine blood test (Mahshid et al., 2022), providing crucial information for therapy and assessment of treatment efficacy. Safe delivery to the inner ear, moreover, might allow the use of contrast agents for precise visualization of inner ear disorder (e.g., endolymphatic hydrops) by imaging. Guidelines will be necessary to establish shared approaches concerning, for example, the optimal volume for sampling/injection, the type of microneedle for a given treatment, the follow-up. Strong collaborative efforts are required between researchers, clinicians, companies, and regulatory agencies to unlock the great potential of precision medicine in the inner ear.
