# Corrigendum: Precision medicine: a new era for inner ear diseases

**DOI:** 10.3389/fphar.2024.1385698

**Published:** 2024-02-27

**Authors:** Elisa Tavazzani, Paolo Spaiardi, Donatella Contini, Giulio Sancini, Giancarlo Russo, Sergio Masetto

**Affiliations:** ^1^ Department of Molecular Medicine, University of Pavia, Pavia, Italy; ^2^ ICS-Maugeri IRCCS, Pavia, Italy; ^3^ Department of Biology and Biotechnology “Lazzaro Spallanzani”, University of Pavia, Pavia, Italy; ^4^ Istituto Nazionale di Fisica Nucleare, Sezione di Pavia, Pavia, Italy; ^5^ Department of Anatomy and Cell Biology, University of Illinois at Chicago, Chicago, IL, United States; ^6^ Department of Medicine and Surgery, University of Milano-Bicocca, Milan, Italy; ^7^ Nanomedicine Center, Neuroscience Center, School of Medicine and Surgery, University of Milano-Bicocca, Milan, Italy; ^8^ Department of Brain and Behavioral Sciences, University of Pavia, Pavia, Italy

**Keywords:** vestibule, cochlea, inner ear, nanoparticles, microneedles, precision medicine

In the published article, there was an error in the **Funding** statement. The CUP numbers were missed. “The author(s) declare financial support was received for the research, authorship, and/or publication of this article. The fee of this publication is supported by the following grants: FIS. SPAIA.PRIN 2022; PRIN 2022 funded by the European Union–Next-Generation EU, PRIN 20228AAJRL; PRIN 2022 funded by the European Union–Next-Generation EU, 2023-ATEQC-0026—multifunctional nanocarriers as advanced biomaterials for targeted and therapeutic RNA delivery [MixnMatch] financed to: SP, MS, and SG respectively” and include original text. The correct Funding statement appears below.

